# Restoration of tumor suppressor miR-34 inhibits human p53-mutant gastric cancer tumorspheres

**DOI:** 10.1186/1471-2407-8-266

**Published:** 2008-09-21

**Authors:** Qing Ji, Xinbao Hao, Yang Meng, Min Zhang, Jeffrey DeSano, Daiming Fan, Liang Xu

**Affiliations:** 1 State Key Laboratory of Cancer Biology and Institute of Digestive Diseases, Xijing Hospital, Fourth Military Medical University, Xi'an, Shaanxi 710032, PR China; 2 Department of Radiation Oncology, Comprehensive Cancer Center, University of Michigan, Ann Arbor, MI 48109, USA

## Abstract

**Background:**

MicroRNAs (miRNAs), some of which function as oncogenes or tumor suppressor genes, are involved in carcinogenesis via regulating cell proliferation and/or cell death. MicroRNA miR-34 was recently found to be a direct target of p53, functioning downstream of the p53 pathway as a tumor suppressor. miR-34 targets Notch, HMGA2, and Bcl-2, genes involved in the self-renewal and survival of cancer stem cells. The role of miR-34 in gastric cancer has not been reported previously. In this study, we examined the effects of miR-34 restoration on p53-mutant human gastric cancer cells and potential target gene expression.

**Methods:**

Human gastric cancer cells were transfected with miR-34 mimics or infected with the lentiviral miR-34-MIF expression system, and validated by miR-34 reporter assay using Bcl-2 3'UTR reporter. Potential target gene expression was assessed by Western blot for proteins, and by quantitative real-time RT-PCR for mRNAs. The effects of miR-34 restoration were assessed by cell growth assay, cell cycle analysis, caspase-3 activation, and cytotoxicity assay, as well as by tumorsphere formation and growth.

**Results:**

Human gastric cancer Kato III cells with miR-34 restoration reduced the expression of target genes Bcl-2, Notch, and HMGA2. Bcl-2 3'UTR reporter assay showed that the transfected miR-34s were functional and confirmed that Bcl-2 is a direct target of miR-34. Restoration of miR-34 chemosensitized Kato III cells with a high level of Bcl-2, but not MKN-45 cells with a low level of Bcl-2. miR-34 impaired cell growth, accumulated the cells in G1 phase, increased caspase-3 activation, and, more significantly, inhibited tumorsphere formation and growth.

**Conclusion:**

Our results demonstrate that in p53-deficient human gastric cancer cells, restoration of functional miR-34 inhibits cell growth and induces chemosensitization and apoptosis, indicating that miR-34 may restore p53 function. Restoration of miR-34 inhibits tumorsphere formation and growth, which is reported to be correlated to the self-renewal of cancer stem cells. The mechanism of miR-34-mediated suppression of self-renewal appears to be related to the direct modulation of downstream targets Bcl-2, Notch, and HMGA2, indicating that miR-34 may be involved in gastric cancer stem cell self-renewal/differentiation decision-making. Our study suggests that restoration of the tumor suppressor miR-34 may provide a novel molecular therapy for p53-mutant gastric cancer.

## Background

MicroRNAs (miRNAs) are a conserved class of non-coding 20–22 nt small RNAs that regulate gene expression by binding to mRNA, leading to mRNA degradation or inhibition [1]. miRNAs regulate a variety of biological processes, including developmental timing, signal transduction, tissue differentiation and maintenance, disease, and carcinogenesis [1]. Emerging evidence demonstrates that miRNAs also play an essential role in stem cell self-renewal and differentiation by negatively regulating the expression of certain key genes in stem cells [1]. One study has shown that MicroRNA-21 knockdown disrupts glioma growth *in vivo *and displays synergistic cytotoxicity with neural precursor cell delivered S-TRAIL in human gliomas [2]. Another recent study shows that miRNA Let-7 regulates self renewal of breast cancer stem cells [3]. Other miRNAs, such as miR-15 and miR-16, have been reported to be able to downregulate Bcl-2, a proto-oncogene overexpressed in many type of cancers, leading to a prevention of apoptosis [4,5]. It has been shown that this obstacle to apoptosis due to overexpression of Bcl-2 results in an increased number of stem cells *in vivo *[6]. This suggests that apoptosis plays a role in regulating the microenvironments of stem cells [7]. Therefore, the Bcl-2 signaling pathway is necessary for the survival of stem cells, especially cancer stem cells, because of the overexpression of Bcl-2 in cancer cells.

Recently, miRNA miR-34 was identified as a p53 target and a potential tumor suppressor [4,8-12]. Over 50% of human cancers have mutant p53 and the expression of miR-34a, b, c appears to be correlated with p53 [10,12]. Bommer et al. reported that the abundance of the three-member miRNA34 family is directly regulated by p53 in cell lines and tissues, and the Bcl-2 protein is regulated directly by miR-34 [10]. The expression of miR-34 is dramatically reduced in 6 of 14 (43%) non-small cell lung cancers (NSCLC) and the restoration of miR-34 expression inhibits growth of NSCLC cells [10]. He et al. reported that ectopic expression of miR-34 induces cell cycle arrest in both primary and tumor-derived cell lines, which is consistent with the observed ability of miR-34 to downregulate a program of genes promoting cell cycle progression [12]. miR-34a has been reported to be involved in p53-mediated apoptosis in colon cancer and pancreatic cancer [8,9]. Tazawa et al. provided evidence that miR-34a induced senescence-like growth arrest in human colon cancer [13]. Taken together, these published studies establish that miR-34 is a new tumor suppressor functioning downstream of the p53 pathway, and provide impetus to explore the functional restoration of miR-34 as a novel therapy for cancers lacking p53 signalling.

It has been reported that miR-34 targets Notch, HMGA2, and Bcl-2, genes involved in the self-renewal and survival of cancer stem cells [10,12,14]. Delineating the role of miR-34 in regulation of cell growth and tumor progression, as well as its potential relationship to cancer stem cells, will help us better understand the p53 tumor suppressor signalling network, facilitate our research in carcinogenesis and cancer therapy, and serve as a basis for our exploration of novel strategies in cancer diagnosis, treatment, and prevention. Thus far, there is limited study on miRNA and gastric cancer; the link between p53 downstream target miR-34 and gastric cancer has not been established; and the role of miR-34 in gastric cancer remains to be investigated. In the current study, we examined the effects of functional restoration of miR-34 by miR-34 mimics and lentiviral miR-34a on human gastric cancer cells, and the effect of miR-34 on tumorsphere formation and growth of p53-mutant gastric cancer cells.

## Methods

### Cell culture and reagents

Human gastric cancer cell lines Kato III, AGS, N87, MKN45, and normal human lung fibroblast cell line WI-38 were purchased from American Type Culture Collection and cultured in DMEM (HyClone, Logan, UT), supplemented with 10% fetal bovine serum (FBS; HyClone, Logan, UT). miRNA miR-34a, b, c mimics, antagonists, and negative control miRNA mimic (NC mimic) were obtained from Dharmacon (Chicago, IL) with the sequences for *hsa-miR-34a: *5'-*uggcagugucuuagcugguugu*-3'*; hsa-miR-34b: *5'-*caaucacuaacuccacugccau*-3'*; hsa-miR-34c: *5'-*aggcaguguaguuagcugauugc*-3'. Bcl-2 3'UTR luciferase reporter plasmid or its mutant were kindly provided by Dr. Eric Fearon of the University of Michigan [10].

### miR-34 mimic transfection

Gastric cancer cells were transfected 24 hours after being seeded in 6-well plates. miRNA mimics (100 pmol) in 200 μl of serum-free, antibiotic-free, medium were mixed with 5 μl of Lipofectamine 2000 transfection reagent (Invitrogen, Carlsbad, CA) dissolved in 200 μl of the same medium and allowed to stand at room temperature for 20 min. The resulting 400 μl transfection solutions were then added to each well containing 1.6 ml of medium. Six hours later, the cultures were replaced with 2 ml fresh medium supplemented with 10% FBS and antibiotics. For Western blot, cells were collected after an additional 48 hours.

### Lentiviral miR-34a infection and stable cells

The feline immunodeficiency virus (FIV) lentiviral system expressing miR-34a (miR-34a-MIF) or vector control (MIF), as well as their lentiviral packaging system, were purchased from System Biosciences (SBI, Mountain View, CA). Gastric cancer cells were infected with the FIV lentiviral system expressing miR-34a (miR-34a-MIF) or vector control (MIF), according to the manufacturer's instructions, and stable cells were obtained by antibiotic selection (Zeocin 50 μg/mL, Invitrogen).

### miR-34 Bcl-2 3'UTR luciferase reporter assay

Kato III cells were transfected in 6-well plates with 2 ug of Bcl-2 3'UTR luciferase reporter plasmid or its mutant, and 2 ug of the control β-galactosidase plasmid per well, using Lipofectamine 2000 (Invitrogen). Cells in each well were also co-transfected with 100 pmol of each miR-34 mimics or NC mimic as indicated, using Lipofectamine 2000. Luciferase assays were performed 24 hrs after transfection using Bright-Glo Luciferase Assay System (Promega). Luciferase activity was normalized relative to β-galactosidase activity detected by the β-galactosidase Assay System (Promega). In each case, Mutant Bcl-2 3'UTR indicates the introduction of alterations into the seed complementary sites of Bcl-2 3'UTR [10]. Luciferase activity was normalized relative to β-gal activity (Promega).

### Western blot analysis

To determine the levels of protein expression, cells were harvested and lysed in a RIPA lysis buffer (50 mM Tris-HCl, pH 8.0, 150 mM NaCl, 0.1% SDS, 1% NP-40, 0.25% Sodium deoxycholate and 1 mM EDTA) with freshly added protease inhibitor cocktail (Roche) for 15 min on ice, then centrifuged at 13,000 rpm for 10 min. Whole cell extract was measured for total protein concentration using Bradford reagent (Bio-Rad, Hercules, CA), and proteins were resolved by SDS-PAGE (Bio-Rad). After electrophoresis, the proteins were electrotransferred to nitrocellulose membranes (Bio-Rad), blocked with 5% skimmed milk, probed with the relevant primary antibody followed by HRP (horseradish peroxidase) conjugated secondary antibody (Pierce, Rockford, IL), and detected with the SuperSignal West Pico chemiluminescence substrate (Pierce). Intensity of the desired bands was analyzed using TotalLab software (Nonlinear Dynamics, Durham, NC).

### Quantitative real-time PCR (qRT-PCR)

Quantitative real-time PCR was performed to determine the expression levels of potential miR-34 target genes. 24 hours after miR-34 mimic transfection of Kato III cells (100 pmol per well in 6-well plates), potential target genes' mRNA levels were measured by qRT-PCR with TaqMan SYBR Green PCR System (Applied Biosystems). Briefly, total RNA was extracted from the transfected cells using TRIZOL (Invitrogen) according to the manufacturer's instructions. Reverse transcription was performed using a TaqMan Reverse Transcription Kit (Applied Biosystems). For qRT-PCR, 1 μl of gene primers with SYBR Green (Applied Biosystems) in 20 μl of reaction volume was applied. Primers were designed as: Bcl-2, forward, 5'-CAT GCT GGG GCC GTA CAG-3', reverse, 5'-GAA CCG GCA CCT GCA CAC-3'; HMGA2, forward, 5'-TTT GTA ATC CCT TCA CAG TCC-3', reverse, 5'-TTT CTC ACC CGC CCA C-3'; Notch1, forward, 5'-ATC CAG AGG CAA ACG GAG-3', reverse, 5'-CAC ATG GCA ACA TCT AAC CC-3'; Notch2, forward, 5'-GGA CCC TGT CAT ACC CTC TT-3', reverse, 5'-CAT GCT TAC GCT TTC GTT TT-3'; Notch3, forward, 5'-TGA TCG GCT CGG TAG TAA TG-3', reverse, 5'-CAA CGC TCC CAG GTA GTC A-3'; Notch4, forward, 5'-TGC GAG GAA GAT ACG GAG TG-3', reverse, 5'-CGG GAT CGG AAT GTT GG-3'; β-actin, forward, 5'-ATG CAG AAG GAG ATC ACT GC-3', reverse, 5'-TCA TAG TCC GCC TAG AAG CA-3'. All reactions with TaqMan Universal PCR Master Mix (Applied Biosystems) were performed on the Mastercycler *realplex *2 system (Eppendorf, Westbury, NY). Target gene mRNA levels were normalized to Actin mRNA according to the following formula: [2^ - (C_T_^target ^- C_T_^Actin^)] × 100%, where C_T _is the threshold cycle. Fold increase was calculated by dividing the normalized target gene expression of the treated sample with that of the untreated control, with the value from the NC mimic set as 1.

### Cell cycle analysis

For cell cycle analysis by flow cytometry, Kato III cells were transfected with miR-34 mimics or NC mimic in 6-well plates, trypsinized 24 hours later and washed with phosphate-buffered saline, and fixed in 70% ethanol on ice. After centrifugation, cells were stained with 50 μg/ml propidium iodide and 0.1 μg/ml RNase A, and analyzed by flow cytometry using a FACStar Plus™. Each histogram was constructed with data from at least 5,000 events. Data were analyzed to calculate the percentage of cell population in each phase using CellQuest software (Becton Dickinson).

### Caspase-3 activation assay

Caspase activation of transfected Kato III cells was determined following the instructions of a Caspase-3 activation assay kit (BioVision, Mountain View, CA). 24 hours after transfection, cells were lysed and whole cell lysates (20 μg) were incubated with 25 μM fluorogenic substrate DEVD-AFC in a reaction buffer (containing 5 mM DTT) at 37°C for 2 h. Proteolytic release of AFC was monitored at λex = 405 nm and λem = 500 nm using a fluorescence microplate reader (BMG LABTECH, Durham, NC). Relative increase of fluorescence signal was calculated by dividing the normalized signal in each treated sample with that of NC mimic as 100.

### Cell cytotoxicity assay

For cytotoxicity assay, the water-soluble tetrazolium salt WST-8 [2-(2-methoxy-4-nitrophenyl)-3-(4-nitrophenyl)-5-(2,4-disulfophenyl)-2H-tetrazolium, monosodium salt] uptake method was employed, using CCK-8 reagent (Dojindo, Gaithersburg, MD). Briefly, Kato III cells were transfected with miR-34 mimics or NC mimic for 24 h, plated in 96-well plates (5,000 cells/well), and treated with serially diluted chemotherapeutic agents in triplicate. After 96 h incubation, 20 μl/well CCK-8 reagent was added and incubated at 37°C for 1–3 h. Optical density was measured at 450 nm and 650 nm using a microplate reader (BMG LABTECH). Final absorbance was obtained by subtracting the absorbance at 450 nm from that at 650 nm, and cell viability (%) was normalized by dividing final absorbance of treated samples with that of the untreated control. IC_50_, the drug concentration that inhibits 50% cell growth, was calculated by GraphPad Prism 5.0 (San Diego, CA), as we described previously [15].

### Tumorsphere culture

Cells were suspended in serum-free culture medium DMEM containing 1% N2 supplement, 2% B27 supplement, 1% antibotic-antimycotic (Invitrogen), 20 ng/ml human FGF-2 (Sigma, Saint Louis, MO), and 100 ng/ml EGF (Invitrogen), and plated in 24-well ultra-low attachment plates (Corning, Corning, NY) at 2,000 cells per well. 7–10 days later, plates were analyzed for tumorsphere formation and were quantified using an inverted microscope (Olympus) at 100×, 200×, and 400× magnification. For subsequent quantification of cell numbers per tumorsphere, tumorspheres were collected and filtered through a 40 um sieve (BD Biosciences, San Jose, CA), and disassociated with 2.5% trypsin, while the viable cells were counted with trypan blue exclusion.

### Statistical analysis

Data were analyzed with Student's two-tailed *t*-test or one-way ANOVA, using GraphPad Prism 5.0 software (GraphPad Prism, San Diego, CA). *P *< 0.05 was defined as statistically significant.

## Results

### Expression of miR-34 and target genes in human gastric cancer cell lines

**Figure 1:**
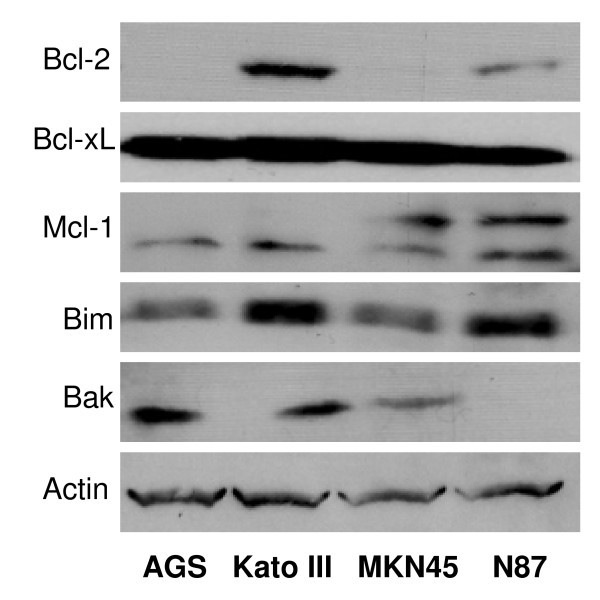
Western blot analysis of the expression of Bcl-2 family proteins in human gastric cancer cell lines.

**Figure 2:**
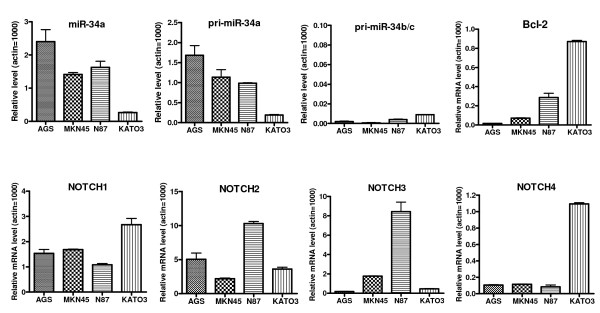
**qRT-PCR analysis of the relative expression levels of miR-34s and target genes in human gastric cancer cell lines.** The cells were lyzed to extract total RNA for qRT-PCR. Data were normalized to that of Actin and the relative levels are shown (Actin = 1000).

We have examined the four human gastric cancer cell lines, Kato III, AGS, N87, and MKN45, for their expression levels of Bcl-2 as well as other members of the Bcl-2 family proteins. As shown in Figure 1, Kato III cells have a high level of Bcl-2, while AGS and MKN-45 cells have very low levels of Bcl-2 (undetectable by Western blot). We next examined these gastric cancer cell lines for the expression level of miR-34 and target genes using qRT-PCR. Kato III cells have the lowest levels of both pri-miR34a and mature miR-34a, and the highest expression levels of target genes Bcl-2, Notch1, and Notch4 (Figure 2). Since miR-34 is a downstream target of the p53 pathway and Bcl-2 is a direct target of miR-34, our data with Kato III are consistent with the cells' p53-mutant status, i.e., Kato III has mutant p53, the lowest level of miR-34, and the highest level of Bcl-2. Therefore, we focused on this cell line for the current study of the effect of miR-34 restoration.

### Transfection of miR-34 mimics in p53-mutant gastric cancer cells

**Figure 3:**
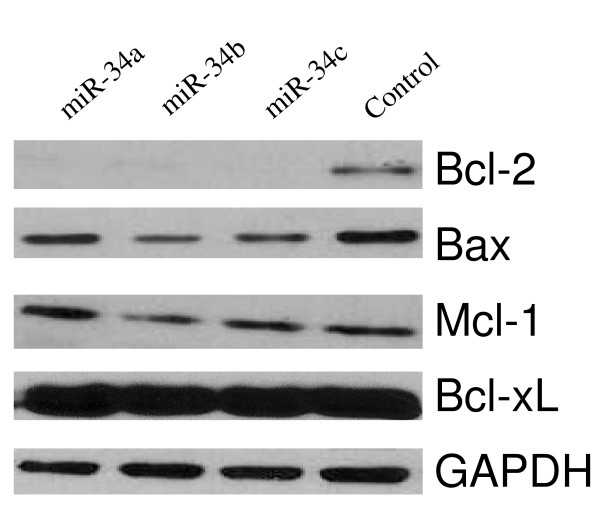
**Restoration of miR-34 by miR-34 mimic transfection downregulates target gene Bcl-2 expression.** Western blot analysis of the potential miR-34 target protein Bcl-2 48 hours after miR-34 mimic transfection of Kato III cells (100 pmol per well in 6-well plates).

**Figure 4:**
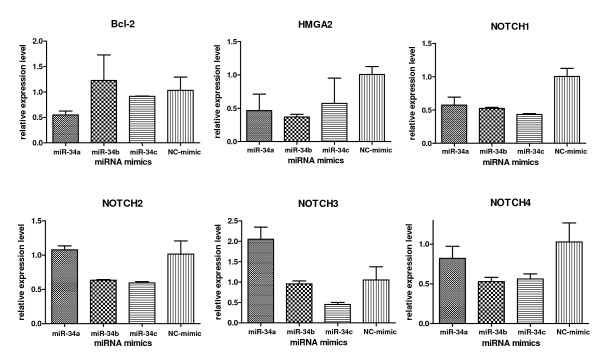
**Quantitative real-time PCR shows that restoration of miR-34 by miR-34 mimic transfection downregulates target gene expression.** 24 hours after miR-34 mimic transfection of Kato III cells (100 pmol per well in 6-well plates), potential target gene mRNA levels were measured by qRT-PCR with SYBR Green PCR system (TaqMan). The comparative threshold cycle CT method was used to calculate relative gene expression levels compared with actin, then normalized with the value from NC mimic as 1.

For miR-34 restoration, we transfected the Kato III cells with miR-34 mimics. As shown in Figure 3, Western blot analysis revealed that transfection of miR-34 mimics downregulated target gene Bcl-2 expression at the protein level, but had no obvious effect on Bcl-xL and Mcl-1 expression, indicating that the Bcl-2 knockdown by miR-34 mimics was sequence-specific. As a target of miR-34, Bax was also downregulated by miR-34. Western blot results were validated by qRT-PCR analysis (Figure 4). More importantly, other potential miR-34 target genes were inhibited in addition to Bcl-2. As shown in Figure 4, Notch1 and HMGA2 were inhibited by all three miR-34a, b, c mimics, while miR-34b mimic inhibited Notch2 and 4, and miR-34c mimic inhibited Notch1-4. Notch1-2 knockdown by miR-34 mimics has been confirmed by Western blot (data not shown).

### Transfected miR-34s are functional

**Figure 5:**
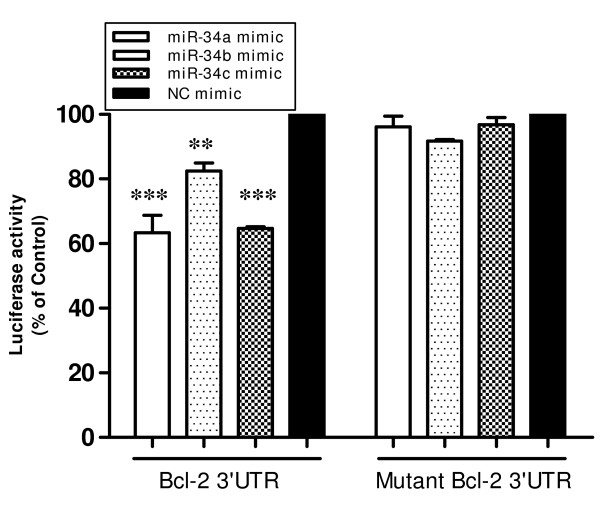
**Bcl-2 3'UTR Luciferase Reporter Assay shows that the miR-34 mimics transfected are functional.** KATO3 cells were transfected in all wells of 6-well plates with 2 ug of Bcl-2 3'UTR luciferase reporter plasmid or its mutant, and 2 ug of the control β-galactosidase plasmid per well. Cells in each well were also co-transfected with 100 pmol of each miR-34 mimic or NC mimic as indicated, using Lipofectamine 2000. Luciferase activity was normalized relative to β-gal activity. Data are presented as mean ± s.e. **P < 0.01, ***P < 0.01, versus that of negative control miRNA mimic (NC mimic), two-tailed t-test, n = 3.

To evaluate whether the transfected miR-34 mimics were functional, we carried out the Bcl-2 3'UTR reporter assay as described by Bommer et al. [10]. KATO3 cells were transfected with Bcl-2 3'UTR luciferase reporter plasmid or its mutant, plus the control β-galactosidase plasmid and 100 pmol of each miR-34 mimic or NC mimic. As shown in Figure 5, the transfected miR-34 mimics effectively inhibited luciferase reporter gene expression, which is controlled by Bcl-2 3'UTR in the promoter region. However, mutation in the Bcl-2 3'UTR complimentary to the miR-34 root sequence abolished this effect, indicating that the observed reporter activity is miR-34 sequence-specific. The results demonstrate that the transfected miR-34a, b, c are functional, and confirm that Bcl-2 is a direct target of miR-34, consistent with earlier reports [8,10,16].

### miR-34 restoration results in Kato III cell accumulation in G1 phase and caspase-3 activation

**Figure 6:**
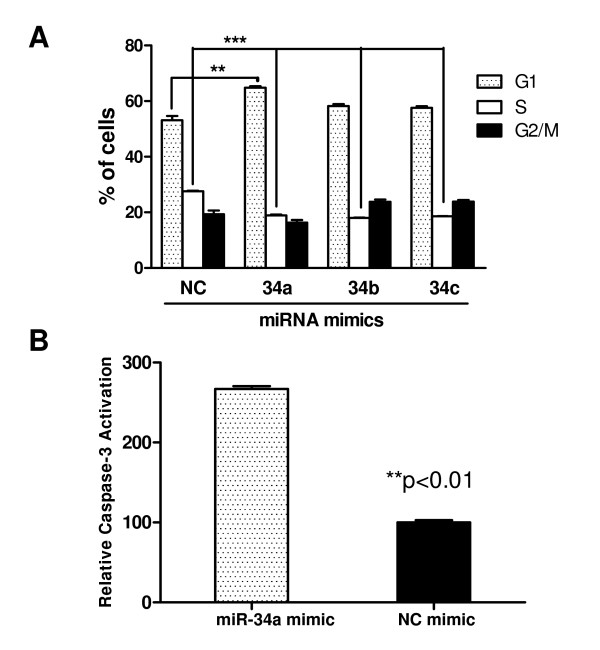
**Restoration of miR-34 in Kato III cells resulted in G1 block and caspase-3 activation.** A. Cell cycle analysis of Kato III cells after miR-34 restoration. Cell cycle analysis was performed 24 hours after transfected with miR-34 mimics or negative control mimic (NC mimic). Cells were stained with propidium iodide after ethanol fixation and analyzed by flow cytometry. **P < 0.01, ***P < 0.001, versus that of NC mimic, one-way ANOVA, n = 2. B. Caspase-3 activation in Kato III cells after miR-34 restoration. 24 hours after transfection, cells were lysed for measurement with the Caspase-3 activation assay kit (BioVision). Relative increase of fluorescence signal was calculated by dividing the normalized signal in each treated sample with that of NC mimic as 100. **P < 0.01 versus that NC mimic, two-tailed t-test, n = 3.

After validating that the transfected miR-34 mimics were functional, we carried out cellular assays to examine the effects of miR-34 restoration on gastric cancer cells. First we evaluated the effect of miR-34 mimics on cell cycle. As shown in Figure 6A, the miR-34 mimics induced an accumulation of Kato III cells in G1 phase and a reduction of cells in S phase, consistent with other reports on miR-34 restoration in various tumor models [4,8,10,12,13,16,17]. This effect on cell cycle is similar to that of p53 restoration as we previously reported [18-23], indicating that miR-34 restoration can restore p53 signalling, at least in part, in the cells lacking a functional p53 pathway. Since part of the p53 tumor-suppressing function is via promoting apoptosis [19,20], we next examined the effect of miR-34 restoration on apoptosis. As shown in Figure 6B, transient transfection of miR-34 mimics resulted in significantly increased caspase-3 activation, a key indication of the cells undergoing apoptosis.

### miR-34 restoration chemosensitizes gastric cancer cells with a high level of Bcl-2

**Figure 7:**
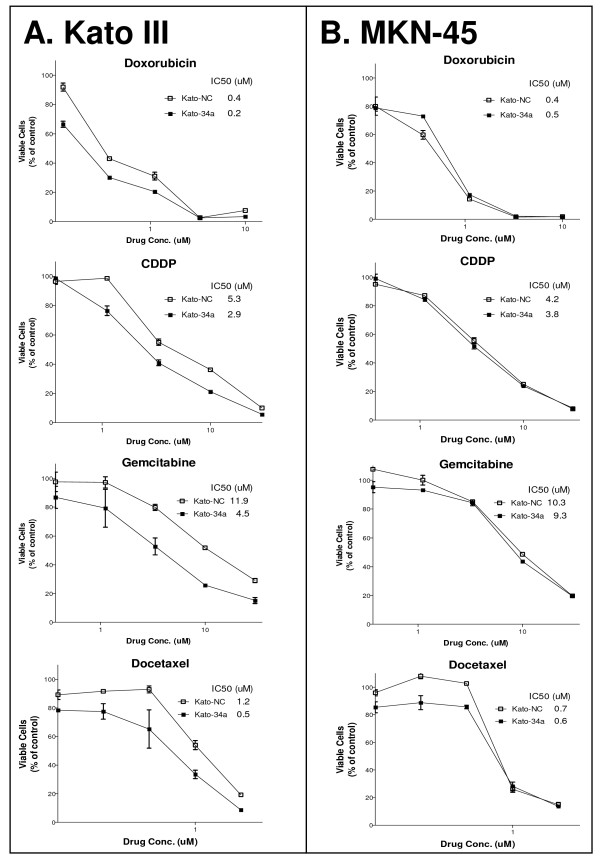
**Restoration of miR-34 chemosensitizes Kato III cells with high Bcl-2, but not MKN-45 cells with low Bcl-2.** MTT-based cytotoxicity assay of Kato III (**A**) and MKN-45 (**B**) cells transfected with miR-34a mimic or control mimic. The cells were transfected with miR-34a or NC mimics by Lipofectamine 2000. 24 hr later, the cells were trypsinized and plated in 96-well plates and treated with the indicated chemotherapeutic agents in triplicate. The MTT-based WST-1 assay was carried out on Day 4. Restoration of miR-34a in Kato III cells with high Bcl-2 rendered the cells 2-3-fold more sensitive to chemotherapies, but had no effect on MKN-45 cells with low Bcl-2. 34a, miR-34a mimic; NC, non-specific control miRNA mimic.

Bcl-2 is a key inhibitor of apoptosis and protects cancer cells from apoptosis induced by chemotherapeutic agents [24,25]. Bcl-2 is a direct target of miR-34, and our data have shown that miR-34 restoration inhibits Bcl-2 expression. We therefore investigated whether miR-34 restoration could sensitize gastric cancer cells with high a level of Bcl-2 to chemotherapy. We chose four chemotherapeutic agents, doxorubicin, cisplatin (CDDP), gemcitabine, and docetaxel, all of which are used in gastrointestinal cancer chemotherapy. As shown in Figure 7A, miR-34 restoration in Kato III cells rendered the cells 2-3-fold more sensitive to the four chemotherapeutic agents, as compared with the cells transfected with the control mimic, based on IC50 data. We have also used siRNA specific to Bcl-2 in these cytotoxicity assays and observed a similar, 2-3-fold chemosensitization in the Kato III cells transfected with Bcl-2 siRNA (data not shown). However, for gastric cancer MKN-45 cells that have a low level of Bcl-2 and a high level of miR-34, miR-34 restoration showed no chemosensitization (Figure 7B). The same results were observed with Bcl-2 siRNA transfection (data not shown). Our data demonstrate that miR-34 restoration can chemosensitize those gastric cancer cells that have high levels of Bcl-2 and low basal levels of miR-34, which are dependent on Bcl-2 for survival and drug resistance.

### miR-34 restoration inhibits gastric cancer cell growth

**Figure 8:**
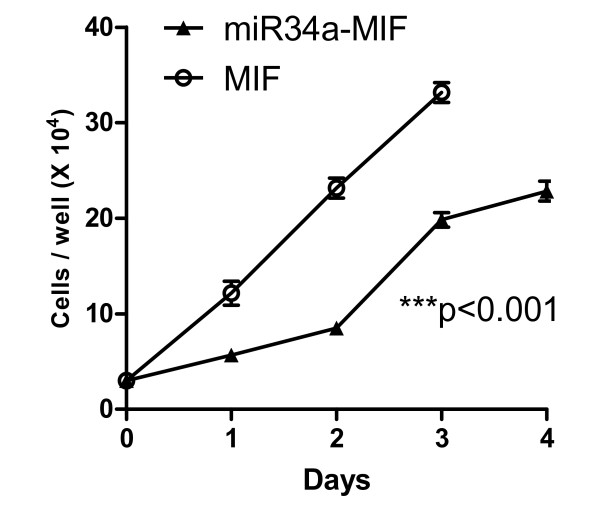
**Restoration of miR-34 in Kato III cells delays cell growth.** Kato III cells were infected with feline immunodeficiency virus (FIV) lentiviral system expressing miR-34a (miR-34a-MIF) or vector control (MIF), and stable cells were obtained by antibiotic selection (Zeocin 50 μg/mL) and validated for miR-34a expression. The stable cells were plated in a 24-well plate with equal cell density. Cells in triplicate were collected by trypsinization and viable cells were counted by Trypan Blue exclusion at 24-h intervals over 4 days, using a Coulter cell counter (Beckman). ***P < 0.001 versus MIF vector control, two-way ANOVA, n = 3.

To evaluate the long-term effects of miR-34 restoration, we have employed a lentiviral system to express miR-34a and have generated stable cells. Briefly, Kato III cells were infected with the feline immunodeficiency virus (FIV) lentiviral system expressing miR-34a (miR-34a-MIF) or vector control (MIF), according the manufacturer's instructions, and stable cells were obtained by antibiotic selection (Zeocin 50 μg/mL, Invitrogen). For cell growth analysis, the stable cells were plated in a 24-well plate with equal cell density. Cells in triplicate were collected by trypsinization, and viable cells were counted by Trypan Blue exclusion at 24 hour intervals over 4 days using a Coulter cell counter (Beckman, Fullterton, CA). As shown in Figure 8, lentiviral restoration of miR-34a in Kato III cells significantly delayed cell growth (*P *< 0.001, n = 3), a biological activity similar to that of the p53 restoration we reported previously [19-22].

### miR-34 restoration inhibits gastric cancer tumorspheres

In our attempts to isolate gastric cancer stem cells, the multiple cell surface markers we used did not provide robust, tumorsphere-forming, tumor-initiating cells or tumor stem cells. Since no cellular markers for gastric cancer stem cells have been widely accepted thus far, in the current study we employed tumorsphere culture to explore whether there is any link between miR-34 and tumorsphere-forming cells.

**Figure 9:**
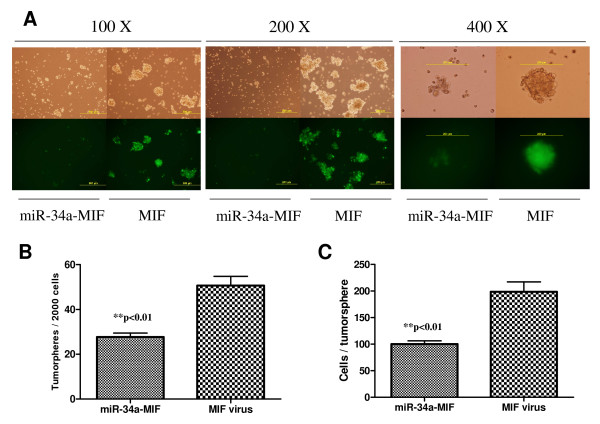
**Restoration of miR-34 by MIF lentiviral system inhibits Kato III tumorspheres.** Kato III cells infected by miR-34a MIF or control vector MIF were plated for tumorsphere formation as described in *Materials and Methods*. 7–10 days later, tumorspheres were observed under microscope (**A**) and quantified (**B**). **C**. Quantification of cell numbers per tumorsphere. Tumorspheres were collected and filtered through a 40 um sieve (BD Biosciences), and dissociated with trypsin for single cell suspension. Cells were counted with trypan blue exclusion and the data are presented as number of cells per tumorsphere. **P < 0.01, Student's *t*-test, n = 3.

Tumorsphere culture, in which cells grow in suspension under non-adherent culture conditions to become tumoroid spheres, has been widely used to assess the self-renewal potential of stem cells and cancer stem cells [3,14,26,27]. Briefly, in our study the stable clones from Kato III cells infected with miR-34a-MIF or control vector MIF were plated for tumorsphere culture in ultra-low adhesion plates. 7–10 days later, tumorspheres were observed under a microscope and quantified. As shown in Figure 9, restoration of miR-34 by MIF lentiviral system inhibited Kato III tumorsphere formation and growth; the stable cells with functional miR-34a restoration had significantly fewer tumorspheres, and the formed tumorspheres were significantly smaller, as compared with that of the MIF control (*P *< 0.001, Student's *t*-test, n = 3). This result was confirmed by a separate tumorsphere study in a 96-well based single cell tumorsphere culture, in which the tumorspheres were seen to be from single cells, not from cell aggregates. Our data provide the first evidence that miR-34 is able to inhibit tumorsphere formation and growth in p53-mutant gastric cancer cells, implying that miR-34 might play a role in the self-renewal of gastric cancer cells, presumably gastric cancer stem cells.

## Discussion

Our studies demonstrate that miR-34 is involved in the p53 tumor suppressor network; restoration of miR-34 is able to re-establish the tumor-suppressing signalling pathway in human gastric cancer cells lacking functional p53. More significantly, miR-34 potently inhibits tumorsphere formation and growth in p53-mutant human gastric cancer cells, providing the first proof-of-concept that there is a potential link between the tumor suppressor miR-34 and gastric cancer cell self-renewal, which involves the presumed gastric cancer stem cells. The mechanism of miR-34-mediated suppression of gastric cancer cell self-renewal might be related to the direct modulation of downstream targets Bcl-2, Notch, and HMGA2, implying that miR-34 may be involved in gastric cancer stem cell self-renewal/differentiation decision-making. Our data suggest that miR-34 may hold significant promise as a novel molecular therapy for human gastric cancer, potentially for gastric cancer stem cells.

Proteins in the Bcl-2 family are central regulators of programmed cell death, and members that inhibit apoptosis, such as Bcl-2 and Bcl-xL, are overexpressed in many cancers and contribute to tumor initiation, progression, and resistance to therapy [28]. Bcl-2 is the founding member of this family of proteins and was first isolated as the product of an oncogene [29,30]. This family of proteins includes both anti-apoptotic molecules, such as Bcl-2 and Bcl-xL, and pro-apoptotic molecules such as Bax, Bak, Bid, and Bad [31]. These are crucial regulators of apoptosis mediated by the Bcl-2 family of proteins [29,32,33]. Overexpression of Bcl-2 is observed in a majority of human cancers, including gastric cancer [34]. The expression level of Bcl-2 protein also correlates with resistance to a wide spectrum of chemotherapeutic agents and radiation therapy [30,35-37]. Overexpression of Bcl-2 protein decreases the pro-apoptotic response to such cellular insults as irradiation and chemotherapy, leading to resistance to the treatments [38-40]. Thus, Bcl-2 is a highly attractive target for the development of novel molecular therapy for the treatment of human cancer.

In our recent study with multidrug resistant human gastric cancer AGS cells, Bcl-2 upregulation and p53 downregulation are found to be involved in drug resistance [34]. Thus, simultaneous inhibition of Bcl-2 function and restoration of p53 represents a promising strategy to overcome drug resistance and improve efficacy for the treatment of p53-mutant gastric cancer. This strategy was explored in the current study, where p53 downstream target miR-34 was restored in p53-mutant gastric cancer Kato III cells with a high level of Bcl-2 and low levels of miR-34, resulting in downregulation of Bcl-2 and Notch/HMGA2, tumor cell growth inhibition and accumulation in G1 phase, and chemosensitization and Caspase-3 activation/apoptosis. miR-34 restoration could thus rebuild, at least in part, the p53 tumor-suppressing signalling network in gastric cancer cells lacking p53 function. This multi-mode action of miR-34 provides a therapeutic advantage over other molecular therapies, in that miR-34 has multiple targets and can work on multiple cell signalling pathways simultaneously, leading to synergistic effects that may translate into improved clinical efficacy for gastric cancer patients with p53 deficiency and multidrug resistance.

Another important implication from the current study is that our data provide a potential link between tumor suppressor miR-34 and the presumed gastric cancer stem cells. Cancer stem cells are a small subpopulation of cells capable of self-renewal and differentiation, and have been identified in a variety of tumors [14,41-45]. Cancer stem cells are believed to be responsible for tumor initiation, progression, metastasis, and resistance to therapy [26,46-49]. To be maximally effective, cancer therapy must be directed against both the resting cancer stem cells and the proliferating cancer cells [47]. This may be possible if specific stem cell signals are inhibited using molecular therapy, while at the same time proliferating cells are attacked by conventional therapies [48,50]. There are limited studies of gastric cancer stem cells, and thus far there are no suitable, widely accepted cellular markers for these rare cells [51,52]. Stem cells are defined by their ability to undergo self-renewal, as well as by multi-lineage differentiation [53]. In our attempts to isolate gastric cancer stem cells, the multiple cell surface markers we used in gastric cancer cell lines did not provide robust, tumorsphere-forming, tumor-initiating cells or cancer stem cells. This might be due to the use of incorrect markers, or the fact that primary tumor tissues should have been examined instead of the established cell lines. However, our data indicate that miR-34 restoration inhibits tumorspheres from p53-mutant gastric cancer cells, suggesting that miR-34 might be involved in the self-renewal of the presumed gastric cancer stem cells. In a separate project on pancreatic and prostate cancer stem cells, our preliminary data indicate that miR-34s are indeed involved in these cancer stem cells, supporting our hypothesis (Ji et al., manuscript submitted). Currently, we are testing a series of cellular markers for human gastric cancer stem cells (CD44, CD24, CD133, ESA, ALDH1, etc.) and using human primary gastric cancer tissues to identify the true side population of the assumed gastric cancer stem cells, and to delineate the role of miR-34 in these tumor-initiating cells.

## Conclusion

Our results demonstrate that in p53-deficient human gastric cancer cells, restoration of functional microRNA miR-34 inhibits cell growth, induces apoptosis, and leads to chemosensitization, indicating that miR-34 may restore, at least in part, the p53 tumor-suppressing function. miR-34 restoration inhibits tumorsphere formation and growth, which is reported to be correlated to the self-renewal of cancer stem cells. The mechanism of miR-34-mediated suppression of self-renewal might be related to the direct modulation of downstream targets Bcl-2, Notch, and HMGA2, indicating that miR-34 may be involved in gastric cancer stem cell self-renewal/differentiation decision-making. Our study suggests that restoration of the tumor-suppressor miR-34 may provide a novel molecular therapy for p53-mutant gastric cancer.

## Competing interests

The authors declare that they have no competing interests.

## Authors' contributions

QJ participated in the design of the study, carried out the molecular and cell studies, and helped draft the manuscript. XH and JD participated in the tumorsphere assays and performed the statistical analysis. YM helped with Western blot and cytotoxicity assay. MZ helped with qPCR. DF participated in the design of the study. LX conceived of the study, participated in its design and coordination, and drafted the manuscript. All authors read and approved the final manuscript.

## Pre-publication history

The pre-publication history for this paper can be accessed here:


